# Cluster randomized evaluation of Adolescent Girls Empowerment Programme (AGEP): study protocol

**DOI:** 10.1186/s12889-017-4280-1

**Published:** 2017-05-05

**Authors:** Paul C. Hewett, Karen Austrian, Erica Soler-Hampejsek, Jere R. Behrman, Fiammetta Bozzani, Natalie A. Jackson-Hachonda

**Affiliations:** 1 Population Council, 4301 Connecticut Avenue, Washington, D.C. 2008 USA; 2 Population Council, P.O. Box 17643-00500, Nairobi, Kenya; 3 Population Council, One Dag Hammarskjold Plaza, New York, NY 10017 USA; 4 Independent consultant, 08019 Barcelona, Fluvia 101, 3o 4a Spain; 5 Departments of Economics and Sociology, University of Pennsylvania, 3718 Locust Walk, Philadelphia, P.A. 19104 USA; 6 London School of Hygiene and Tropical Medicine, 15-17 Tavistock Place, London, WC1H 9SH UK; 7 Population Council, Plot 3670, No. 4, Mwaleshi Road, Olympia Park, 10101 Lusaka, Zambia

**Keywords:** Adolescent girls, Randomized trial, Multi-sectoral, Zambia, Empowerment, Savings, Financial education, Nutrition education, Sexual and reproductive health, HIV, HSV-2

## Abstract

**Background:**

Adolescents in less developed countries such as Zambia often face multi-faceted challenges for achieving successful transitions through adolescence to early adulthood. The literature has noted the need to introduce interventions during this period, particularly for adolescent girls, with the perspective that such investments have significant economic, social and health returns to society. The Adolescent Girls Empowerment Programme (AGEP) was an intervention designed as a catalyst for change for adolescent girls through themselves, to their family and community.

**Methods/design:**

AGEP was a multi-sectoral intervention targeting over 10,000 vulnerable adolescent girls ages 10–19 in rural and urban areas, in four of the ten provinces of Zambia. At the core of AGEP were mentor-led, weekly girls’ group meetings of 20 to 30 adolescent girls participating over two years. Three curricula ― sexual and reproductive health and lifeskills, financial literacy, and nutrition ― guided the meetings. An engaging and participatory pedagogical approach was used. Two additional program components, a health voucher and a bank account, were offered to some girls to provide direct mechanisms to improve access to health and financial services. Embedded within AGEP was a rigorous multi-arm randomised cluster trial with randomization to different combinations of programme arms. The study was powered to assess the impact across a set of key longer-term outcomes, including early marriage and first birth, contraceptive use, educational attainment and acquisition of HIV and HSV-2. Baseline behavioural surveys and biological specimen collection were initiated in 2013. Impact was evaluated immediately after the program ended in 2015 and will be evaluated again after two additional years of follow-up in 2017. The primary analysis is intent-to-treat. Qualitative data are being collected in 2013, 2015 and 2017 to inform the programme implementation and the quantitative findings. An economic evaluation will evaluate the incremental cost-effectiveness of each component of the intervention.

**Discussion:**

The AGEP program and embedded evaluation will provide detailed information regarding interventions for adolescent girls in developing country settings. It will provide a rich information and data source on adolescent girls and its related findings will inform policy-makers, health professionals, donors and other stakeholders.

**Trial registration:**

ISRCTN29322231. March 04 2016; retrospectively registered.

**Electronic supplementary material:**

The online version of this article (doi:10.1186/s12889-017-4280-1) contains supplementary material, which is available to authorized users.

## Background

This paper reviews the design and methods of a multi-arm, randomized cluster evaluation of the Adolescent Girls Empowerment Programme (AGEP) in Zambia. The programme was designed as a multi-sectoral intervention targeted to vulnerable, rural and urban adolescent girls ages 10–19. Embedded in the design of the programme was a rigorous, randomized evaluation that recruited a cohort of eligible adolescent girls invited to participate in the programme and girls residing in control areas. The evaluation sample was interviewed at baseline in late 2013 and early 2014 and is being tracked annually through 2017, with mid-term results collected in 2015 immediately after the end of the intervention and endline results in 2017, two years post programme termination [1].

Adolescent girls in less developed countries face a variety of risks and challenges in achieving positive and successful transitions to adulthood [2]. In Zambia, a significant proportion of girls enter marriage and/or begin childbearing early, even prior to the expected age of school leaving. As indicated by the 2013–2014 Zambia Demographic and Health Survey (DHS), nearly one in three girls aged 20–24 had married by age 18, with a similar percentage having begun childbearing [1]. The prevalence in rural areas is nearly double that of urban areas for both early marriage (39% versus 22%) and early childbearing (42% versus 21%) [3]. Not surprisingly, given rates of early marriage and childbearing, the risk of school leaving for girls during the school age years is high, with 18% of primary school age girls and 59% of secondary age girls not currently attending school [1]. The Zambia DHS also indicates that girls in Zambia begin sexual activity early, have low rates of condom use and have risk behaviours that increase their chances of acquiring sexually transmitted infections (STIs), including HIV. For instance, the median age of sexual debut is 17.7 years, approximately 1 year prior to marriage, and the prevalence of sexual initiation among 15–19 year olds is 27%. Further, of those who are currently engaging in premarital sexual activity, only 36% reported using a condom at last sex. The low rate of consistent condom use implies exposure to STIs, with 4% of adolescents 15–19 reporting having an STI or symptoms of a sexually transmitted disease and 5% having acquired HIV. The prevalence of HIV more than doubles by the time girls reach the ages of 20–24 years [1].

The literature indicates that women and adolescent girls in many less developed settings are lacking the needed assets and capacities required to make more positive and healthier transitions that would facilitate their breaking out of persistent poverty and closing the economic and livelihoods gap between men and women. In such settings, women and girls’ opportunities are inhibited by traditional practices, adverse gender norms and roles, and weak institutions and laws [4, 5]. For instance, Duflo argues that inequality, poverty and the lack of access to economic assets, opportunities and labour markets are primary drivers for the persistent disadvantage of women relative to men [5] Other studies have directly linked gender power inequality to HIV risk behaviours and exposures [6]. Women and girls who have limited social capital and are more isolated engaged in riskier sexual behaviours, are more likely exposed to sexual violence and less likely to be tested for HIV [7, 8].

**Fig. 1:**
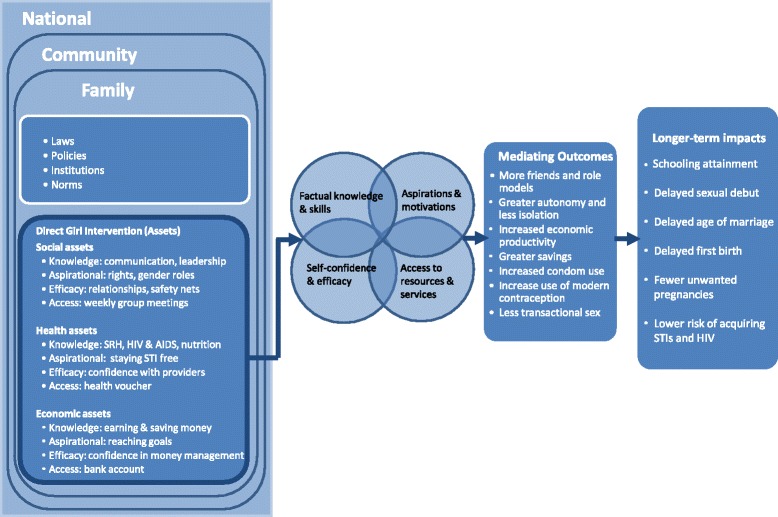
AGEP Theory of Change

To address these disadvantages, the Population Council, working in collaboration with the YWCA, Making Cents International, the National Savings and Credit Bank of Zambia (Natsave) and the Zambian Ministry of Health designed AGEP, a multi-sectoral and girl-centered intervention. The theory of change underlying AGEP (Fig. 1) proposed that working directly with adolescent girls to build their economic, health and social assets would facilitate positive change across a broad set of critical adolescent girls’ experiences and outcomes, including early marriage and first birth, schooling attainment, age of sexual debut, sexual risk behaviour, and transmission of sexually transmitted infections, including HIV. The theory underlying AGEP defines assets as a store of value that can be drawn upon by adolescent girls to address challenges and overcome vulnerabilities [9–12]. It was posited that an asset-building framework operates by enhancing girls’ knowledge and capacities, building confidence and efficacy, strengthening social networks, changing aspirations, and providing access to both health and financial services and resources. Further, it was theorized that such changes at the individual-level would percolate up to changes in the contextual environment within the family and community.

## Intervention design

The goal of AGEP was to reach a minimum of 10,000 adolescent girls ages 10–19 across ten sites in four of the ten provinces of Zambia; half of the sites were urban areas and half rural areas. Provinces were purposefully selected in collaboration with the donor, weighing feasibility, resources for the programme and evaluation, and the objective of representing vulnerable adolescent girls. As the programme was specifically designed to reach the most-vulnerable adolescent girls, high-density urban housing compounds were targeted, while a more general sample of rural residential communities were targeted.

### Girls group meetings

The AGEP intervention operated for a period of two years in each site from late 2013 and early 2014 through late 2015 and early 2016. Implementation of the programme was initiated sequentially by site, after the baseline evaluation survey was completed in each site. The core component of AGEP was group meetings in which 20 to 30 girls met at a local community space for one to two hours per week, most often on weekends. A community-based model was selected as school-based sexual reproductive health programs have been known to have limited impact because teachers find it hard to break out of their typical teaching styles and programs are adversely affected by an already low quality of schooling [13, 14]. In addition, school-based programs exclude out-of-school girls, which were meant to be included in the intervention. The girls’ groups were stratified by age (10–14 and 15–19) and by marital/fertility status; girls stayed with their original groups as they aged or otherwise changed status. The group sessions were based on three core curricula (Table 1) and used illustrative vignettes, role play and participatory methods to maximize impact. The meetings were led by an older, young woman from the community who had been trained to initiate and guide educational sessions and exercises on a variety of subjects. The mentors were trained in groups at central locations at the beginning of the programme, with one refresher training approximately one year into the program. Monthly mentor meetings also took place with site coordinators to address mentor questions, experiences and provide short reviews of curriculum topics.

**Table 1:** AGEP core curricula implemented through weekly girls’ groups meetings

Health and life skills	Session topics
Introductory sessions (9 sessions)	What to expect (×2 sessions); teamwork; gender roles; communication; self-esteem; goal identification; goal setting; personal relationships
Reproductive health (9 sessions)	Life cycle; body changes; pregnancy; avoiding unintended pregnancy; reproductive myths; sexual desire; unsafe abortion, abortion and stigma; maternal mortality
Life skills (11 sessions)	Healthy relationships; reasons for delaying sex; strategies for delaying sex; passive, assertive and aggressive behaviour; drugs and alcohol; peer-pressure; decision-making; communications; managing emotions; conflict resolution
HIV, AIDs and STIs (6 sessions)	Information on transmission; myths and facts; HIV testing and counselling; risky behaviour; relationship of STIs and HIV; Stigma and discrimination
Gender and gender-based violence (4 sessions)	Sexual exploitation; avoiding and reporting sexual violence; rape and gender violence; preventing unwanted advances
Leadership (2 sessions)	Defining and identifying the qualities of leadership; community service and action
Human rights (3 sessions)	Defining human and children’s rights; sexual and reproductive health rights; HIV and AIDS and human rights.
Financial education	
Dreams (1 session)	Strengths, weakness, opportunity and threats in achieving dreams
Saving and earning money (10 sessions)	Why save, choose a savings goal, make a savings plan, banks and bank accounts, options for earning money, risky ways to earn money, difference between needs and wants, controlling spending, planning income and expenses, saving regularly
Managing money (7 sessions)	Safe places to save, dealing with setbacks in saving, own versus others money, talking about money, do’s and don’ts when talking about money, resolving conflicts over money, resolving conflicts role play
Good money management (1 session)	Journey to good money management
Nutrition	
Building blocks of nutrition (3 sessions)	Nutritional needs of adolescent girls (types of foods), role of food in the body (dietary diversity), anaemia–causes and symptoms
Nutrition in pregnancy, infancy & early childhood (3 sessions)**†**	Nutrition needs during pregnancy, infant feeding, child feeding and growth monitoring

*Note:* Two additional sessions developed for the end of the programme that helped girls to plan for “life after AGEP”. There was also an additional session for girls in Arm 3 providing details about the savings account

^**†**^denotes sessions only for girls who were aged 15 and older at baseline.

All the girls who were assigned to the intervention were invited to participate in AGEP girls’ groups. Two additional programme components were randomly assigned to some of the girls groups. The additional components were: 1) the provision of a health voucher and, 2) an offer to open a bank savings account. Eligibility for these add-on components was determined by whether or not the girl group was located in a cluster that was randomized to receive the additional components of the intervention. The assignment of girls to the programme component is discussed further in the evaluation design section below.

### Health vouchers

Girls who attended AGEP in randomly-selected areas to receive a health voucher targeted to improve access to ten general health and sexual and reproductive health services at partner public and private health providers (Table 2). The design and implementation of the AGEP health voucher was done in consultation with the Ministry of Health at the national, provincial and district level [15]. A published review of the literature found that health vouchers had been successful at increasing service utilization and improving the quality of health services provision across a variety of health care and developing country settings. The review, however, noted that the breadth and scientific quality of evidence was limited and more rigorous assessments of effectiveness were needed [16]. A study of a programme that provided health vouchers specifically to adolescent girls in Nicaragua found that it directly reduced many of the barriers to adolescent sexual reproductive health care, including improving provider knowledge around adolescent health issues, attitudes and communication practices [17]. These studies suggested the potential for improving SRH care among adolescents in Zambia and the value of implementing a voucher programme embedded within a rigorous evaluation design.

**Table 2:** Services provided with health voucher

1	General physical exam and other non-sexual reproductive health services
2	Family planning consultation and method
3	Pregnancy testing and consultation
4	STI consultation and treatment for girl and her partner
5	HIV counselling, testing and referral
6	Antenatal care and laboratory testing
7	Comprehensive abortion care
8	Consultation for other sexual reproductive health issues
9	Cervical cancer screening
10	Gender-based violence care and referral

*Note*: Availability of services varied somewhat as not all facilities were able to provide the full list of services

In addition to the sexual and reproductive, nutritional and general health knowledge building during the weekly girls’ groups, two additional programme elements supported the AGEP health voucher component: 1) the training of health facility staff in providing adolescent girl-friendly health services, and 2) a results-based financing of service provision at health facilities [18]. All participating health facility providers and staff were given a two-day training on adolescent girl-friendly health services that followed a set curriculum specifically designed for AGEP and supported by the Ministry of Health. Monthly monitoring and quality assurance visits to health facilities were also conducted [19]. The training sessions provided an overview of AGEP and the mechanisms of the health voucher, but also covered topics geared to improve the quality of service delivery for adolescents, including values clarification about family planning, HIV and STIs, communicating and counselling adolescents, identifying and addressing barriers to adolescent health care and the sexual and reproductive health rights of adolescents.

The results-based financing component of the health voucher also provided a basis from which to reimburse the health facility and provider for service use among adolescent girls. At private and non-governmental organization facilities, AGEP reimbursements were based on negotiated fees for each service provided. At public facilities, as health services are generally free, the payments to the facilities for each service, whose rates were agreed on with MoH and were uniform for all public facilities, were distributed by fixed percentages: to the providers (50%), to the health facilities for supplies (25%), for per-diems to the district health staff to assist in monitoring service provision (20%), and to the District Health Office for overall management of the programme (5%). Reimbursements were a function of the total number of girls with vouchers obtaining services and the number of services that they accessed during the period that the vouchers were usable. It should be noted that unlike other programme elements, due to delays in finalizing the arrangements, the health voucher started after one year of the programme and continued one year after the AGEP girls groups ended.

### Savings accounts

While the girls groups provided capacity building in money management, budgeting and savings, the provision of a bank account was designed to provide a mechanism for knowledge and skills to be operationalized in practice. The bank account, provided to girls who would not otherwise typically have access to financial services, was hypothesized to reinforce girls’ money management skills, promote economic asset building, grow a culture of savings, facilitate economic independence and provide assets in cases of emergencies or other basic needs. Savings accounts, as opposed to micro-credit, were used as studies had shown in other settings limited results for such programs [20] and that micro-credit was not an appropriate first exposure to formal financial services for vulnerable adolescents [21]. Savings accounts, when appropriately designed for adolescents, had been shown in other contexts to improve financial literacy, increase the self-efficacy and savings behaviours [22]. Even in resource poor environments among vulnerable populations, savings accounts were found to increase savings and improve positive opinions about HIV prevention methods [23].

The bank account component was implemented in partnership with the National Savings and Credit Bank of Zambia (NatSave). The AGEP “Girls Dream” savings account provided adolescent girls a formal place to store money. The girls who were eligible for the savings account were provided an orientation in the girls’ group sessions and a field trip was organized to the nearest NatSave branch to provide additional information and to open accounts. Girls who were under the age of 18 years were required to have co-signatories present at the account opening. The bank account was specifically tailored to the financial needs of the girls, with a very low minimum opening balance of KW 2.5 (US $0.50) and the ability to deposit or withdraw funds with no fees. The girls who opened accounts could deposit on their own, although for girls under age 18 years the accounts required their co-signatories to withdraw funds. While no direct financial resources in the form of grants or loans were provided to AGEP girls, market research based on focus group discussions conducted during the pilot period suggested that adolescent girls had a variety of sources of income that may be used to build their savings, including cash from parents, paid piecemeal work, agricultural production, selling goods made and transactional sex [24].

## Intervention population

As noted previously, AGEP was designed to serve a minimum of 10,000 vulnerable adolescent girls in Zambia aged 10 to 19 in ten sites located within four of the ten provinces of Zambia. The study provinces and the number of sites per province were selected purposefully through discussions with the donor regarding representation of the target population and consideration of the feasibility of operating AGEP while also conducting a randomized evaluation. Study sites within provinces were selected randomly from a sampling frame of potential sites, anchored to proximally-located health and banking facilities. The sampling frames of potential sites for urban and rural areas were developed separately. For urban areas, a list of high-density residential compounds was created, and sites selected randomly from the list, while in rural areas sites were randomly selected among areas that met the following conditions: that they were located within 15 km from a health facility and within 65 km of a NatSave bank branch.

AGEP was also designed to draw participants who expressed multiple levels of vulnerability through their residential location and the socio-economic characteristics of their households. To avoid over-representing girls in AGEP who have already experienced the outcomes that were to be prevented by the programme (early marriage, becoming pregnant, dropping out of school), these indicators were not directly used to identify vulnerability. Instead, an indicator of vulnerability was created using whether girls were behind in school for their age as a proxy, which also permits characterization of the vulnerability of girls younger than those who already have been married, pregnant or dropped out of school. Conceptually, the measure captures adolescents who are at risk of adverse outcomes, given the primacy of education in a child’s life at these ages. Early in the school-going process, many children fall behind in school due to late entry, repetition of grades, and temporary withdrawal from school; all of these events are a reflection of some degree of personal and household vulnerability. The information used to develop the measure of vulnerability was collected along with other household socio-economic characteristics via a household survey in all study clusters, which was subsequently used as a sampling frame to determine eligible girls for participation in the programme with the most vulnerable girls selected from the list to receive invitations to participate.

## Evaluation methods and design

The objective of the AGEP evaluation was to implement a rigorous randomized design to more confidently attribute the changes in girls’ lives to their participation in AGEP. It was expected that the programme would have a positive impact on the longer-term demographic, reproductive and health outcomes of participating adolescents. It was also expected that the impact of the programme on these longer-term outcomes would be mediated through a set of individual assets or skills that the girls acquire through the programme. Given that the AGEP evaluation has multiple components that may lead to improved outcomes, the study was designed and statistically powered to evaluate the impact of each of these components on adolescent girl outcomes, independently for rural and urban areas.

### Randomised cluster design

The AGEP evaluation was designed as a multi-arm randomised cluster trial where clusters within the ten study sites were randomised to receive different combinations (arms) of AGEP. The experimental and control arms of the study are displayed in Table 3, along with their associated components. Clusters assigned to the first intervention arm were assigned only the weekly girls’ group sessions. Clusters in the second intervention arm were assigned the health voucher in addition to the weekly girls’ group sessions, while clusters in the final intervention arm were assigned all components, including the bank account. The multi-arm evaluation provided for a rigorous assessment of the marginal benefit, as well as the incremental cost-effectiveness of each additional component of the intervention. All girls selected for participation in AGEP within a cluster were assigned to the intervention that was randomly selected for that cluster; girls in clusters for the control arm received neither interventions nor placebo exposures.

**Table 3:** Randomisation arms of AGEP

Study arm	AGEP components
Intervention arm 1	Weekly girls group mentor-led sessions
Intervention arm 2	Weekly girls group mentor-led sessions + health voucher
Intervention arm 3	Weekly girls group mentor-led sessions + health voucher + bank account
Control	No components of the AGEP intervention

A cluster in the AGEP evaluation was delineated by Census Supervisory Areas (CSAs) that were compiled by the Zambia Central Statistical Office (CSO) for the 2010 national census. CSAs contained approximately 750 households in urban areas and 300 households in rural areas, although the average number of households varies considerably as population densities vary geographically. CSAs in urban areas were spatially relatively small, perhaps a few hundred metres long and wide, while CSAs in rural areas encompassed multiple kilometres. The assignment of CSAs to the experimental and control arms was conducted through a random selection process at a public lottery. The public lottery was conducted to maximize the transparency and community acceptance of AGEP. Local political and community leaders were invited to participate in the lottery, conducted at a centrally-located public facility. The lottery was conducted via a two-step selection process in which a CSA was first randomly selected for participation among all CSAs in the site and then an AGEP arm assigned through a second random selection process.

### Study outcomes

The overall objective of AGEP was to make a meaningful change in the traditionally observed trajectories of vulnerable adolescent girls in Zambia as they pertained to early family formation, low schooling attainment and poor sexual and reproductive health. The process of change was posited to advance in phases, with the more immediate changes in the girls’ assets and empowerment deriving from their participation in AGEP, translating into positive behavioural change and, ultimately, to longer-term change over the course of four years in which empowered adolescents would realize more positive transitional outcomes. As the concept of empowerment, mediating and longer-term outcomes are potentially broad and multifaceted, a set of representative indicators was used to epitomise their operationalization in the evaluation as noted in Table 4. The indicators of empowerment ranged across the broad domains of social, economic and health and included measures of self-efficacy, social connections, positive gender normative beliefs, financial literacy, access to savings and sexual and reproductive health knowledge, among others. The longer-term indicators of programme impact ranged across three domains, socio-demographic, educational and health.

**Table 4:** Primary and secondary study outcome indicators and measures

Immediate programme outcomes	Asset domain
Percentage of girls with high self-efficacy	Social
Percentage of girls with strong role models and social support	Social
Average number of friends, friends who can be counted on	Social
Percentage of girls who hold positive gender normative believes	Social
Average score on financial literacy scale	Economic
Percentage of girls who have opened a formal bank account	Economic
Average score on knowledge of sexual reproductive health	Health
Average number of modern contraceptives known	Health
Percentage having comprehensive knowledge of HIV	Health
Percentage of girls accessing sexual and reproductive health services	Health
Mediating outcomes indicators	
Percentage of girls working for cash or in-kind in the past year	Economic
Percentage of girls with a modest amount of savings	Economic
Percentage of girls who have used a condom during last sex with a non-marital/non-cohabiting partner	Sexual and reproductive health
Percentage of girls who are using modern contraception	Sexual and reproductive health
Percentage of girls who have engaged in transactional sex	Sexual and reproductive health
Longer-term outcomes indicators	Outcome domain
Percentage of girls ever married	Socio-demographic
Percentage of girls ever pregnant and given birth	Socio-demographic
Percentage of girls experiencing unwanted pregnancy	Socio-demographic
Percentage of girls completing grade 7 and grade 9	Educational
HIV prevalence among girls	Sexual and reproductive health
Herpes Simplex Virus Type-2 (HSV-2) prevalence among girls	Sexual and reproductive health

### Hypotheses

The study will test several hypothesis to assess the impact of AGEP and its components. The hypothesis tests reflect the causal pathways as illustrated in the theory of change (Fig. 1). The impact of the intervention is posited to have operated through four general and domain-specific mechanisms, 1) by improving factual knowledge and building skills, 2) increasing self-confidence and efficacy, 3) increasing adolescent aspirations and motivations and, 4) improving access to key resources and services. The first set of hypotheses reflect the expectation that AGEP is having a strong and positive effect on the array of assets that girls can build upon and utilize to affect change. These are measured as immediate programme outcomes indicators in Table 4. A second set of hypotheses is made regarding the impact of the intervention on what are conceptualized here as mediating factors, reflected through HIV and pregnancy risk reduction behaviours, improved attendance in school and possessing greater economic resources and opportunities. The indicators used to reflect these hypotheses are presented as mediating outcomes indicators in Table 4. Finally, the overall objective and ultimate goal, of the programme is to improve the wellbeing of adolescent girls, reflected in specific ways, by reducing early marriage, early pregnancy and births, increasing educational attainment and reducing the acquisition of HIV and the Herpes Simplex Virus Type – 2 (HSV-2).

#### Weekly girls groups meetings

As noted above, all three intervention arms included weekly meetings in which girls met with a mentor and each other to engage with a curriculum topic for the week, generally discuss their experiences and to socialize with each other. Given the randomized design, it is possible to assess the impact of the girls groups alone. The weekly girls’ group meeting is hypothesized, in particular, to have positive effects on the girls’ sexual and reproductive health, financial and nutritional knowledge, as well as build their communications, money management and self-advocacy skills. The group meetings also targeted the girls’ social assets, including the number of friends reported, the quality of those relationships and whether they report a role model in their lives. Participation in the groups is also hypothesized to reduce social isolation and provide access to community spaces. These relationships and opportunities are, in-turn, hypothesized to weaken regressive gender norms and roles, move aspirations, and improve mediating behaviours, such as attending school. In the domain of schooling attainment, these changes are hypothesized to improve school participation, performance and, ultimately, attainment. If confirmed, these hypothesizes will result in strong and positive relationships between the indicator of randomization to the girls group only study arm and the indicators noted along this continuum of outcomes.

#### Health voucher

As with the bank account component, the health voucher intervention is hypothesized to directly increase access to health facilities and resources, as well as to increase efficacy and decision-making control over the utilization of health facilities and resources. The effect of the heath voucher component is expected to be distinguishable empirically over and above what is realized through the weekly girls groups alone. In the immediate term, the health voucher is hypothesized to increase the use of sexual and reproductive health services, including the use of modern contraception. Improved sexual and reproductive health, e.g., by obtaining counselling, care and treatment for STIs, is hypothesized to reduce HIV acquisition in the longer-term, while access to contraception is hypothesized to reduce unwanted pregnancy and birth outcomes. In general, it is hypothesized that as specific health issues are prevented or resolved, girls will have more positive assessment of their overall health.

#### Bank accounts

As noted in Table 1 above, the weekly girls’ group curriculum includes 19 sessions on setting financial goals, budgeting and managing savings and expenditures. While the lessons themselves are hypothesized to directly increase knowledge and improve financial and entrepreneurial aspirations, the bank account component of AGEP was designed to provide a direct link and access to financial services to catalyse change. It is hypothesized, therefore, that the provision of a bank account will have positive impact on informal and formal savings behaviour. Increased access and control over economic resources is hypothesized to reduce the need for adolescent girls to rely on household resources from which they have less control to obtain personal items such as clothing and hygiene products, schooling supplies and/or pay school fees. Access to bank accounts is also hypothesized to depress the need for adolescent girls to rely on older and potentially riskier partners and exchange-based sexual relationships. These changes are hypothesized to reduce the prevalence of sexual exchange and, ultimately, reduce girls’ acquisition of sexually transmitted infections, including HIV and HSV-2.

### Research population and sample sizes

Participants for the research study were randomly selected among all invited AGEP participants who were never married at baseline, stratified by site and age group. For comparability, selection of the control girls followed a parallel process for identifying and selecting vulnerable girls, specifically a ranking of girls by their vulnerability score and randomly selecting girls below a set threshold, with the threshold determined by the total number of girls needed for recruitment into the programme at each site. As the ultimate objective of the funder and the programme was to improve the well-being of adolescent girls, the longer-term outcomes in Table 4 served as the focal indicators for the sample size calculations. As limited evidence was available for a sample size determination based on known effect sizes, minimally detectable effects were estimated using Optimal Design Plus Software Version 3.0 for a multi-site randomized trial to determine the most efficient sample size (number of clusters and respondents per cluster) given the budget available for fieldwork [25]. Sample sizes were estimated for the evaluation after four years, with conservative estimates of attrition, 20% for the younger cohort and 35% for the older cohort. The samples were stratified by the younger and older cohorts (10–14 and 15–19) and urban and rural. The age stratification was necessary as there was no age mixing of groups in the weekly girls groups and certain aspects of the curricula were adjusted for age-appropriateness by cohort.

The minimally detectable effect sizes were estimated for comparison of each programme component against the control; estimates of effect sizes were not obtained for comparisons between the intervention arms themselves as little prior information was available to guide such an estimation approach. Given a total of ten study sites, it was determined that a total of 40 clusters per arm (four clusters per arm per site) and 20 participants per cluster was the most efficient combination. Baseline (control) estimates of study indicators by age group and residential status were obtained from the 2007 Zambia Demographic and Health Survey [26]. Statistical power of 0.80 and an alpha coefficient of 0.05 were set, while the effect size variability was fixed at 0.00, as analyses would control for site fixed effects [25]. Table 5 provides estimates of prevalence in the control sample and minimal detectable effect sizes, by outcome and study stratifications.

**Table 5:** Estimates of prevalence in the control and minimally detectable effect sizes in percentage points (pp) of programme impact by age-cohort and by rural and urban stratifications

	Total^a^		Rural^b^		Urban^b^	
	Younger	Older	Younger	Older	Younger	Older
Ever had sex	40%, ± 10 pp	82%, ± 9 pp	47%, ± 14 pp	89%, ± 11 pp	34%, ± 13 pp	75%, ± 13 pp
Ever married	15%, ± 6 pp	63%, ± 10 pp	19%, ± 11 pp	76%, ± 13 pp	10%, ± 8 pp	48%, ± 14 pp
Ever given birth	16%, ± 6 pp	70%, ± 10 pp	18%, ± 10 pp	83%, ± 13 pp	13%, ± 8 pp	54%, ± 14 pp
Completed grade 7	61%, ± 10 pp	60%, ± 9 pp	43%, ± 14 pp	41%, ± 15 pp	79%, ± 11 pp	85%, ± 9 pp
Completed grade 9	28%, ± 10 pp	38%, ± 10 pp	11%, ± 11 pp	17%, ± 13 pp	45%, ± 15 pp	64%, ± 14 pp
Ever use of modern contraception	15%, ± 8 pp	55%, ± 7 pp	15%, ± 12 pp	55%, ± 14 pp	15%, ± 12 pp	55%, ± 15 pp
HIV prevalence	6%, ± 4 pp	12%, ± 6 pp	6%, n/a ^c^	7%, n/a ^c^	6%, n/a ^c^	19%, ± 10 pp
HSV-2 prevalence	21%, ± 8 pp	26%, ± 8 pp	21%, ± 11 pp	26%, ± 12 pp	21%, ± 11 pp	26%, ± 12 pp

*Notes*: Younger cohort members are expected to be ages 14–18 at endline; older cohort members are expected to be ages 19–23 at endline. All estimates assume alpha of 0.05, power of 0.80, and site fixed effects

^a^For each age cohort estimates are based on: 10 sites, 4 clusters per arm per site (40 clusters per arm), and 10 subjects per cluster

^b^For each age cohort estimates are based on: 5 sites, 4 clusters per arm per site (20 clusters per arm), and 10 subjects per cluster

^c^Not powered at 0.80 to detect programme impact

The sample size calculations indicated that 3200 girls would be needed at the end of the study to assess all key study impact indicators: 800 girls (within 40 study clusters) in each of the three AGEP arms and in the control arm. An additional 400 girls (within 20 clusters) were designated to be sampled for urban areas in nearby high-density components. These girls and clusters were designed to serve as external controls given the potential for contamination between AGEP and control girls within the same site. Never married girls were targeted at baseline as a significant proportion of older girls would have already experienced the key outcomes that were meant to be impacted by the programme. To achieve the target sample of 3600 girls at the end of the study, the sample size was inflated for non-eligibility and non-response at baseline, attrition from the cohort and refusals for biological specimen collection. Thus, the final estimated sample to be collected at baseline was 7200 or 4800 in AGEP clusters, 1600 in internal control clusters and 800 in external control areas.

### Research instruments

A household survey instrument was implemented prior to baseline to determine eligibility for the intervention and for selecting cases in designated control clusters. The household instrument included a roster of all household members with basic socio-demographic information on each member, household asset ownership, housing quality, recent household shocks, access to financial resources and distances to key resources, including schools, roads, health facilities, banks, etc. Comprehensive annual surveys were conducted at baseline and for four subsequent years. The baseline survey is included as an Additional file 1. The surveys instruments measure changes in attitudes, behaviour, transition status, social assets and cognitive skills that may occur over time regarding: educational attainment and schooling transitions; sexual activity, relationship status and sexual partners; marriage and marital dissolution; sexual and physical coercion and violence; gender attitudes, self- efficacy and locus of control; labour force participation and savings behaviour; living arrangements and household resources; mobility and migration; literacy, numeracy and cognitive skills; and ability; financial literacy and knowledge. Surveys conducted after the baseline also include questions of exposure to the intervention to address potential spillover impacts. All questionnaires were translated into Nyanja, Bemba and Kaonde.

Survey instruments were implemented by electronic data capture. Computer-Assisted Personal-Interviewing (CAPI) was used for questions that are non-sensitive. CAPI is a process of data capture in which the interviewer reads the question from a computer screen and enters the participant’s response directly into a handheld or tablet device. For sensitive questions, including sexual behaviour, sexual violence, HIV risk perception, unwanted pregnancy and abortion, Audio Computer-Assisted Self-Interviewing (ACASI) was used. With ACASI the respondent listened with headphones to pre-recorded questions and response categories, while, if desired and if the participant was literate, simultaneously reading the question on the device screen. The participant entered a response by touching a designated number or option. ACASI maximizes confidentiality and privacy of response, as no one could hear or see the question being read, nor the response option selected.

Biological markers were collected from adolescents aged 15 and older at baseline (Round 1), after Round 3 (at the end of AGEP), and Round 5 (at the end of the evaluation). HIV status was determined via capillary blood draws obtained from finger pricks. HIV tests were conducted by trained and certified voluntary counselling and testing (VCT) staff. In accordance with Ministry of Health guidelines, we conducted serial testing using Determine™ and Uni-Gold™ [27]. Both tests have a very high sensitivity (100%) and specificity (>99%) in controlled clinic evaluations, including a controlled laboratory setting in rural Kenya [28]. The respondent was provided the test results. Post-test counseling was guided by National Guidelines for Testing and Counselling [29].

The HSV-2 biological specimens were collected via finger prick. A sample of whole blood was collected and stored in microtainers and laboratory tested. To conduct the laboratory test, serum was derived from the whole blood and the Kalon ELISA assay used, for which the sensitivity and specificity have been found to be high (92% and 98%) respectively in African populations when compared to Western Blot [30]. Indeterminate results were not retested since their prevalence was so low as to make it impractical. After specimen collection all participants were provided information about HSV-2 detection, symptoms, safe sex practices and treatment options. Respondents were provided vouchers with identification numbers to receive their test results at AGEP participating health centers. A validation of the HSV-2 laboratory testing procedures was conducted prior to testing of specimens for the main study to assure the quality of laboratory testing protocols.

### Economic evaluation

The study also includes an economic evaluation to rigorously assess the cost of implementing AGEP, as well as to assess the incremental cost-effectiveness of the add-on components (health voucher and savings account) relative to the girls group intervention alone.. Direct programme costs were collected from AGEP budgets and financial reports and included both start-up and programme delivery costs for the Population Council and its partners. Participant-specific out-of-pocket and indirect costs data for participation in AGEP and uptake of services were also obtained. The relevant costs for these analyses are the real resource costs of delivering the programme services, not including the costs related to project evaluation. A decision analytic model will be constructed to generate estimates of the incremental costs per negative health outcome averted and positive progress achieved on non-health indicators from participating in AGEP. Incremental cost-effectiveness ratios (ICERS) comparing the add-ons to the girls group only intervention will be calculated to assess whether cost-effectiveness varies by study arm.

### Analysis

The primary objective of the evaluation is to assess the impact of AGEP on adolescents’ intermediate empowerment and longer-term outcomes using the above-noted hypothesis as test criteria. Important as well, analysis will be conducted to understand the underlying processes by which change occurred in the key areas of interest. As a first approach for assessing the impact of AGEP on empowerment and longer-term outcomes, an intent-to-treat analysis (ITT) will be conducted using the original randomised assignment to study arm as the primary indicator of impact. The average treatment effect of the programme on the adolescents in clusters randomised to AGEP relative to girls in clusters randomised to the control will be empirically assessed. The analysis will estimate models that are both unadjusted and adjusted for baseline covariates; both approaches accounting for strata and intraclass correlations within clusters. To accommodate for any baseline differences between AGEP and control clusters that may exist in the outcomes, a difference-in-differences (DID) calculation will be made for each indicator. The mid-term assessment after Round 3 focused on the immediate and mediating indicators as primary outcomes, while in the final assessment after Round 5 the mediating and longer-term outcomes will be the empirical focus.

As girls were invited to participate in AGEP, many choose not to do so, intermittently missed the weekly sessions or left the programme before the programme was completed. Therefore a secondary analysis will be conducted that uses a measure of participation intensity as the indicator of impact rather than the study’s random assignment. It is important to recognize the statistical estimation problems that arise from self-selection into AGEP, and the degree of participation in it, with the characteristics of girls predicting both their uptake of and exposure to the programme and key outcomes and behaviours of interest. A two-stage instrumental variables estimation approach will be used in which the first stage will predict programme participation, using the exogenous randomised assignment as the key instrumental variable, observed baseline covariates will also be included. The second stage will then use the predicted participation intensity from the first stage along with baseline covariates to test the relevant hypotheses. Appropriate tests, including F-tests on excluded instruments, the Wald F-statistic, and the Hansen statistic for over-identification will quantitatively assess the credibility of the selected instrumental variable.

## Discussion

AGEP was an intervention whose objective was to improve important adolescent transitions of girls in Zambia, with the expectation that improved outcomes for girls would lead to life-long improvements in the well-being of women and their children. The rigorous randomized evaluation and cohort study to evaluate AGEP outlined in this protocol paper was designed to address information gaps and the weaknesses recognized in the existing literature, including non-randomized study designs, a lack of information on vulnerable and disadvantaged populations, and short-term durations of assessments [31]. As noted in the literature, the benefits from finding out what works, under what conditions and how for adolescents in a setting such as Zambia is large, as demographically the “cohort of young people age 10–24 is the largest in history,” while epidemiologically developing countries face multi-burdens from diseases and are “characterized by high levels of all types of adolescent health problems” [32].

AGEP was a multi-sectoral intervention built off of the core concept of mentor-led, weekly girls’ group meetings whose primary objective was to build and empower girls by 1) enhancing their knowledge and skills, 2) building their self-confidence and efficacy, 3) changing their aspirations, and 4) providing access to services and resources. The AGEP programme is unique in the amount of investment in this effort, with over two years of weekly programme activities devoted to girls’ empowerment across a range of domains, including sexual and reproductive health, financial literacy, life skills and nutrition. AGEP was not, however, limited to the weekly girls groups, as two additional components, a bank account and health voucher, enhanced the potential impact of the group learnings by providing girls direct access to resources that typically did not exist for them or have high barriers to use. The multi-arm randomized evaluation was designed to assess whether each of these programme components provided additional value added in terms of impact.

Determining the impact of AGEP is important in order to provide needed evidence for governments, donors, and other stakeholders to develop policies and programs for adolescent girls. Delineating costs of the programme and the investment needed for added programme components can provide additional critical information about the investment needed to scale the programme and the incremental costs of investments in each intervention component. To achieve this additional aim, the study research protocol included an embedded economic evaluation that collected programme implementation and participant costs. The economic evaluation will provide cost-effectiveness information for each study arm and an opportunity to compare AGEP with alternative programmes that address the needs of adolescent girls in the areas of early marriage and childbearing, education and sexual and reproductive health.

## Additional file

Additional file 1: AGEP Baseline Survey. (XLSX 2922 kb)

## Abbreviations

ACASI: Audio Computer-Assisted Self-Interviewing
AGEP: Adolescent Girls Empowerment Programme
CAPI: Computer Assisted Personal Interviewing
CSA: Census Supervisory Area
DFID: Department for International Development
HIV: Human immunodeficiency virus
HSV-2: Herpes simplex virus, type-2
MOH: Ministry of Health
NATSAVE: National Savings and Credit Bank of Zambia
STI: Sexually transmitted infections
UNZA-REC: University of Zambia, Research Ethics Committee
YWCA: Young Women’s Christian Association
