# Erratum to: cluster randomized evaluation of adolescent girls empowerment Programme (AGEP): study protocol

**DOI:** 10.1186/s12889-017-4647-3

**Published:** 2017-08-01

**Authors:** Paul C. Hewett, Karen Austrian, Erica Soler-Hampejsek, Jere R. Behrman, Fiammetta Bozzani, Natalie A. Jackson-Hachonda

**Affiliations:** 1 Population Council, 4301 Connecticut Avenue, Washington, D.C 2008 USA; 2 Population Council, P.O. Box 17643-00500, Nairobi, Kenya; 3 Population Council, One Dag Hammarskjold Plaza, New York, NY 10017 USA; 4 Independent consultant, Fluvia 101, 3o 4a, 08019 Barcelona, Spain; 5 Departments of Economics and Sociology, University of Pennsylvania, 3718 Locust Walk, Philadelphia, P.A 19104 USA; 6 London School of Hygiene and Tropical Medicine, 15-17 Tavistock Place, London, WC1H 9SH UK; 7 Population Council, Plot 3670, No. 4, Mwaleshi Road, Olympia Park, 10101 Lusaka, Zambia

## Erratum

After publication of the article [1], it has been brought to our attention that some of the statistical data presented regarding the DHS data in the second paragraph of the background section is incorrect. The authors would like to note the following changes.

1. The following statistics were accidentally reversed: "The prevalence in rural areas is nearly double that of urban areas for both early marriage (39% versus 22%) and early childbearing (42% versus 21%)." This sentence should read: "The prevalence in rural areas is nearly double that of urban areas for both early marriage (42% versus 21%) and early childbearing (39% versus 22%)."

2) The statistic on the prevalence of sexual initiation in the following sentence was incorrect: "For instance, the median age of sexual debut is 17.7 years, approximately 1 year prior to marriage, and the prevalence of sexual initiation among 15–19 year olds is 27%. This sentence should read": For instance, the median age of sexual debut is 17.7 years, approximately 1 year prior to marriage, and the prevalence of sexual initiation among 15–19 year olds is 49%.”
